# Changing the Perceived Pain Intensity in Populations With Spinal Cord Injury and With Health Disparities (HAPPINESS): Protocol for a Feasibility Study

**DOI:** 10.2196/82431

**Published:** 2025-11-19

**Authors:** Ann Van de Winckel, Lin Zhang, Kelvin O Lim, Leslie R Morse, Brianna Woodliff, Gladys Maestre, Kelsey Baker

**Affiliations:** 1 Division of Physical Therapy and Rehabilitation Science, Department of Family Medicine and Community Health Medical School University of Minnesota Minneapolis, MN United States; 2 Division of Biostatistics School of Public Health University of Minnesota Minneapolis, MN United States; 3 Department of Psychiatry and Behavioral Sciences Medical School University of Minnesota Minneapolis, MN United States; 4 Department of Physical Medicine and Rehabilitation Miller Medical School University of Miami Miami, FL United States; 5 Institute of Neuroscience School of Medicine University of Texas Rio Grande Valley Harlingen, TX United States

**Keywords:** neuropathic pain, spinal cord injury, Qigong, body awareness, focus groups, protocol, qualitative, feasibility, randomized controlled trial, pain management

## Abstract

**Background:**

Chronic neuropathic pain affects 69% of adults with spinal cord injury (SCI). Medication, as a primary treatment, has insufficient benefits and significant risks for addiction and adverse effects. Of the available mind and body approaches, Qigong is the most accessible for adults with SCI, with evidence for effectiveness in reducing pain. Still, there is insufficient evidence to make recommendations for adults with SCI. Thus, the feasibility of Qigong in SCI needs to be established. We previously demonstrated feasibility in a pilot study, in which 18 non-Hispanic White adults with SCI completed a 12-week remote Qigong program. However, three additional key elements need to be addressed before performing a larger effectiveness study: (1) feasibility and acceptability of Qigong in adults with SCI with health disparities, (2) feasibility of a study design with a control group, and (3) objective outcome measures.

**Objective:**

This National Center for Complementary and Integrative Health (NCCIH) R34 feasibility study—the HAPPINESS (Changing the Perceived Pain Intensity in Populations With Spinal Cord Injury and With Health Disparities) trial—will build upon our prior study to consolidate feasibility with a rigorous protocol. Our aims are (aim 1) to identify facilitators and barriers to participating in a Qigong study through community event meetings, focus groups, and interviews with 40 stakeholders, of whom at least 50% (n=20) have health disparities, and (aim 2) to establish the feasibility of study design and methods through prespecified targets for recruitment and enrollment, feasibility, and acceptability of design and outcomes.

**Methods:**

For aim 1, a total of 40 SCI stakeholders—adults with SCI, caregivers, health care professionals, or other stakeholders who have experience with caring for adults with SCI—will be invited to attend community event meetings, one focus group, or an interview, where they will provide input on facilitators and barriers related to participating in a Qigong study. For aim 2, using a phase I randomized controlled trial design, 40 adults with SCI-related neuropathic pain (with at least 50% [n=20] having health disparities) will be randomized to a 12-week remotely delivered Qigong intervention (practicing at least 3×/week with the Spring Forest Qigong’s “Five Element Qigong Healing Movements” video), or a short daily pain management survey, completed on a smartphone, iPad, or computer + 6-month follow-up. Feasibility benchmarks, as well as patient-reported outcome measures and objective data from the Fitbit Charge 6 watch, will be collected during the study.

**Results:**

The first participant was recruited on August 20, 2024. Study completion is estimated on January 31, 2027.

**Conclusions:**

The study results will facilitate a rigorous structure to design large effectiveness studies and facilitate a clear pathway for researchers to investigate Qigong and other mind and body approaches for whole-person health in adults with chronic or neurological disorders, including those with health disparities.

**Trial Registration:**

ClinicalTrials.gov NCT06140355; https://clinicaltrials.gov/study/NCT06140355

**International Registered Report Identifier (IRRID):**

DERR1-10.2196/82431

## Introduction

### Background

Chronic neuropathic pain impacts up to 69% of the 308,620 adults with spinal cord injury (SCI) [1-3]. This burning, electric, or stabbing pain can be debilitating and interfere with all aspects of life. The primary line of treatment is pain medication, but it often has insufficient benefits and carries uncomfortable side effects or risks for addiction [4,5]. In addition, there is an underrepresentation of minority groups, women, and older adults in SCI rehabilitation clinical trials [6]. For example, 14% of adults with traumatic SCI are of Hispanic origin; yet, there was an underrepresentation (<10%) of Hispanic or Latinx participants in SCI rehabilitation clinical trials in the United States between 2011 and 2020 [6].

Research showing the effectiveness of mind and body approaches for reducing pain in adults with SCI is limited to 7 studies, among which only 3 (on Yoga and Tai Chi) [7-9] looked at pain reduction, without mentioning which type of pain they were investigating [7-13]. One seated Tai Chi program was well-tolerated in adults with SCI (n=26) with reduced pain and a higher emotional and physical well-being after each session, but their weekly in-person group classes had a dropout of 60% [8].

We reported significant neuropathic pain reduction after remotely delivered Qigong in our feasibility pilot study of 18 adults with SCI-related neuropathic pain [14]. Sample sizes in the above-mentioned studies were small. Moreover, only our study [14] included adults with complete and incomplete tetraplegia, and Tsang et al [11] included adults with incomplete tetraplegia, but all others recruited adults with paraplegia only. Despite these promising results, more accessible interventions for adults with tetraplegia and larger studies are needed.

Substantial evidence suggests that mind and body treatments for physical and mental health may be particularly important for high-risk disadvantaged individuals, including racial or ethnic minorities and those with poor access to conventional treatments because of cost [15]. Factors such as offering the interventions in multiple languages, integrating feedback from participants, and designing programs that are compatible with cultural and religious beliefs would further improve the feasibility and acceptability of mind and body treatments and promote personal use [15-18]. Finally, since 2009, with the increasing opioid epidemic, the US military (Department of Defense [DoD] and Veterans Affairs) has made substantial efforts to provide multidisciplinary, holistic treatments to Veterans with chronic pain [19]. Mind and body approaches have helped military Service Members reduce their pain, improve function and self-management, and increase self-efficacy, but to what extent these approaches are used by adults with SCI to relieve neuropathic pain is unknown [19-22].

Of all mind and body treatment options, Qigong is the most accessible for adults with SCI due to the gentle movements, which can be done in standing, sitting, or lying positions, and due to its focus on breathing and body awareness [23-25]. Yet, there is insufficient evidence to make recommendations for Qigong for adults with SCI at this time. Thus, the feasibility of Qigong in SCI needs to be established. Remote delivery of Qigong is feasible for adults with SCI as it eliminates the commonly reported barriers of in-person interventions: fatigue, pain, transportation, and scheduling difficulties [26]. Moreover, our remotely delivered Qigong practice, which can be done standing, sitting, or lying down, aligns active movements to their ability level with kinesthetic imagery, that is, focusing on the feeling of moving the whole body as if in an upright position, because imagery may be an additional way to improve body awareness and reduce pain [14,25,27]. Therefore, adults with high-level tetraplegia after SCI can participate in this study because they can easily practice Qigong in their wheelchairs or lying down. In other studies, the minimum active muscle strength requirement for exercise programs often excludes these people.

Our preliminary data demonstrate the feasibility and acceptability of practicing Qigong for non-Hispanic White adults with SCI-related neuropathic pain with promising results in terms of pain relief, SCI-related symptoms, improved function, and mental well-being after Qigong practice [14,25]. Eighteen participants completed the study (18/21 enrolled; 86% retention), with 100% adherence to all study components, and 12 participants completed the 1-year follow-up. We enrolled a representative sample of women with SCI (6/18, 33%), adults 65+ years of age (7/18, 39%), living in rural areas (6/18, 33%), Veterans (3/18, 17%), adults in socioeconomic distress (12/18, 67%), and below the poverty threshold (3/18, 17%) [6,14,28]. Even though we recruited nationwide, all participants were non-Hispanic White, pointing to the need for more encompassing recruitment strategies. Participants practiced at home, at least 3 times per week for 12 weeks (123 minutes), with a Qigong video accessible via the internet (ie, the same video as proposed below). Our results showed feasibility and adherence to the intervention (138% of the required intensity of Qigong practice, representing 170 minutes) and no study-related adverse events.

However, despite these promising data, three additional key elements need to be addressed before performing a larger phase II effectiveness clinical trial: obtaining data from adults with SCI with health disparities on (1) feasibility and acceptability of participating in a Qigong study, (2) feasibility of the study design (including an active comparison group) and adherence to the interventions, and (3) obtaining objective outcome measures.

### Purpose of This Study

This R34 feasibility study—the HAPPINESS (Changing the Perceived Pain Intensity in Populations With Spinal Cord Injury and With Health Disparities) trial—will expand on our previous study to consolidate the feasibility with a rigorous protocol that addresses those key elements, with the aims listed below.

#### Aim 1: To Identify Facilitators and Barriers to Participating in a Qigong Study in Adults With SCI With Health Disparities

We will hold community event meetings, focus groups, and in-depth interviews with 40 stakeholders, including at least 50% with health disparities (ie, racial or ethnic minorities, Veterans, or living in rural, underserved areas), to identify which sociodemographic, behavioral, cultural, or structural factors facilitate or hinder participation in the study.

#### Aim 2: To Establish the Feasibility of the Study Design and Methods of the HAPPINESS Trial in 40 Adults With SCI (With at Least 50% [n=20] With Health Disparities) Through Prespecified Targets

The targets are as follows: (1) assessing recruitment and enrollment rates and identifying facilitators and barriers to participation; (2) evaluating recruitment rates for adults with complete versus incomplete SCI to determine the ratio of incomplete versus complete SCI for recruitment estimates in the next phase II randomized controlled effectiveness trial; (3) evaluating the feasibility and acceptability of the design by assessing adherence to Qigong practice or 2-minute daily pain management surveys, study satisfaction, and attrition rates in the Qigong and active comparison groups, as well as identifying facilitators and barriers to practicing Qigong at home; and (4) evaluating the feasibility of the data collection by assessing follow-up rates of outcome measures (% completed assessments at each time point, including objective measures, and weekly and monthly pain surveys).

This R34 feasibility study addresses the National Center for Complementary and Integrative Health (NCCIH) High Priority Pain Research (NOT-AT-22-007) using complementary and integrative interventions. The outcomes of this study will facilitate a rigorous structure to design large effectiveness studies and facilitate a clear pathway for researchers to investigate Qigong and other mind and body approaches for whole-person health, including in adults with chronic or neurological disorders, and those with health disparities.

## Methods

### Overall Study Design

For aim 1, we will hold community event meetings (ie, education and listening sessions) in town halls and health care settings for SCI stakeholders to share information about the research and hold listening sessions. We will organize focus groups and in-depth interviews using the Consolidated Framework for Implementation Research in the Twin Cities and in South Texas. We estimate we will have 3-5 attendees if on Zoom, and 5-8 attendees if in-person. We estimate holding 3 in-depth interviews with the most informative SCI stakeholders, to better understand the facilitators and barriers to remotely delivered Qigong practice, and identify which sociodemographic, behavioral, cultural, or structural factors facilitate or hinder participation in the study as a whole, including access to a computer, iPad, or smartphone and internet needed for Qigong practice or pain management through a Qualtrics survey, and remotely delivered questionnaires. If in person, the focus groups and interviews will be held in Minnesota (University of Minnesota–Twin Cities) and South Texas (University of Texas Rio Grande Valley [UTRGV]).

For aim 2, we will organize a feasibility phase I randomized controlled trial design where 40 adults with SCI-related neuropathic pain with at least 50% (n=20) with health disparities (racial or ethnic minorities, Veterans, or adults living in rural, underserved areas) are randomized to (experimental group) a Qigong introduction class and a 12-week remotely delivered Qigong intervention, with a 6-month follow-up, or (active comparison group) they complete daily 2-minute surveys to manage their pain sent through Qualtrics, with a 6-month follow-up.

Randomization will be generated by an independent biostatistician. The outcome assessors and the biostatistician on the study will be blinded to the group allocation. We have registered this clinical trial with the National Institutes of Health (NIH) clinical trials registry. Our feasibility pilot trial follows the guidelines according to the Obesity-Related Behavioral Intervention Trials model [29] and NCCIH [30]. Based on the guidelines, we developed aims, protocol, and the following a priori feasibility benchmarks to rigorously test the methodology and feasibility of the intervention before a larger effectiveness clinical trial is conducted.

Behavioral assessments will be acquired remotely at 3 time points: at baseline, after the 12-week interventions, and at the 6-month follow-up. This 6-month follow-up period will assess behavioral changes and the sustainability of Qigong practice adherence or pain management survey adherence in daily life. In our prior study, participants enjoyed continuing Qigong practice because it helped them manage their pain [14]. Both groups will receive weekly calls during the 12-week intervention period and monthly calls during the 6-month follow-up to assess neuropathic pain (primary outcome) and neuropathic pain medication, including opioid use, adherence to Qigong practice or the pain management surveys, adverse events, illnesses, and use of health care services. Qualitative information will be collected about their adherence, adverse outcomes, if any, perceptions, effects, and satisfaction with the Qigong practice or pain management surveys. Ecological momentary assessments (EMAs), consisting of 2-minute Qualtrics surveys are delivered 4 times per day for 3 weeks at baseline, during the last 3 weeks of the 12-week interventions, and during the last 3 weeks of the 6-month follow-up to identify feasibility of data that can explore causal relationships between variables of mood, activity, sleep, and so on, and neuropathic pain to understand in each individual which variables increase or decrease neuropathic pain. Objective data on sleep, stress, and other information will be collected continuously with the Fitbit Charge 6 wrist device. Fitbit data will also be time-synchronized with EMA data at those 3 time points to evaluate the feasibility of data that can explore causal relationships between physiological data, behavioral data, and neuropathic pain. The study is offered in English and Spanish to increase accessibility to those for whom English may be a barrier.

The SPIRIT (Standard Protocol Items: Recommendations for Interventional Trials) 2013 checklist with recommended items to address in a clinical trial protocol and related documents (Multimedia Appendix 1) and the SPIRIT template of recommended content for the schedule of enrollment, interventions, and assessments (Table 1) are included.

**Table 1:** Scheduling of enrollment, intervention, and assessments.

		Study period			
Timepoint		Enrollment	12-week intervention	6-month follow-up	Close-out
**Enrollment**		Week 0	Week 1-12	Week 13-37	Week 38
	Eligibility screen	✓			
	Informed consent	✓			
	Baseline assessments (Zoom questionnaires)	✓			
	Allocation	✓			
**Interventions**					
	Qigong		✓	✓	
	Pain management survey		✓	✓	
**Assessments**					
	Fitbit Charge 6 smartwatch	✓	✓	✓	✓
	EMA^a^ surveys	✓	✓	✓	
	Zoom questionnaires	✓	✓		✓
	Weekly calls		✓		
	Monthly calls			✓	

^a^EMA: ecological momentary assessment.

### Study Setting

The clinical trial part of the study is completely remotely delivered. The event meetings, focus groups, and interviews can be held in-person or over secure Zoom. The principal investigator (AVdW) will head event meetings, focus groups, and in-depth interviews with SCI stakeholders with emphasis on those with health disparities in Minnesota. The UTRGV team (KB and GM) will support the event meetings, focus groups, and interviews with the Hispanic community in South Texas, and provide the principal investigator (AVdW) with the transcriptions in English or Spanish with translated versions to English of the focus groups and interviews, the latter of which will be reviewed by GM to ensure cultural adaptation. We will purchase the Spring Forest Qigong Center’s “Five Element Healing Movements” video and introduction class for the intervention in English or Spanish. This video will be reviewed during the meetings, focus groups, and interviews. A certified Spring Forest Qigong instructor will teach the introduction class and hold three 1:1 Zoom sessions with the participants who were randomized to the Qigong intervention group.

### Participants and Recruitment

For aim 1, the inclusion criteria are 40 SCI stakeholders, which can be adults with SCI, caregivers, health care professionals, or other stakeholders who have experience with caring for adults with SCI, especially those with health disparities.

The inclusion criteria for aim 2 are persons aged 18+ years, 40 participants with an incomplete or complete SCI of ≥1 year, medically stable, and with ≥4/10 for highest neuropathic pain intensity level on the Numeric Pain Rating Scale [31]; willing to participate in a remote Qigong intervention (from any location with internet connection), fluent in English or Spanish, access to the internet and a computer, iPad, or smartphone. We aim to recruit at least 50% (n=20) of adults with health disparities (ie, racial or ethnic minorities, Veterans, or living in rural, underserved areas).

The exclusion criteria are uncontrolled seizure disorder, cognitive impairment or communicative disability (eg, due to brain injury) preventing them from following directions or from learning, ventilator dependency, major medical complications, pressure ulcers hindering prolonged sitting or lying down, (planning to become) pregnant or planning a major surgery during the study (given the study duration, regular Qigong practice or surveys, and frequent check-ins), regular Tai Chi or Qigong practice in the past 6 months (3×/week or more) or currently engaged in other rehabilitation programs that would influence outcomes.

We estimate recruiting 30%-40% (12-16/40) of adults with complete versus 60%-70% (24-28/40) incomplete SCI based on our prior data in SCI and on reported statistics of 23% (9/40) complete paraplegia, 15% complete tetraplegia (6/40), 22% incomplete paraplegia (9/40), and 40% incomplete tetraplegia (16/40) [32].

We will recruit from all clinical site partners within the Minnesota Regional SCI Model System, which includes hospitals, clinics, rehabilitation centers, long-term care facilities, and nursing homes in the Twin Cities and Mayo Clinic in Rochester. Specifically in the Twin Cities, we have collaborations within the Veterans Affairs (Minnesota), including the Minnesota Paralyzed Veterans, University of Minnesota Medical Center, University of Minnesota Health (M Health) Fairview, Regions Hospital, Courage Kenny Rehabilitation Institute, and Minnesota SCI associations. Health care providers will provide information to potential participants and post fliers.

M Health Fairview has a recruitment system in place where letters are sent from their system directly to patients with SCI and neuropathic pain. Patients who are interested in participating can contact the researchers. We also collaborate with the SCI patient advocacy association United to Fight Paralysis.

In 2019, a total of 530 adults with new SCI were treated in the Twin Cities clinics, with 311 receiving outpatient therapy in rehabilitation centers. In 2022, a total of 285 adults with SCI received annual evaluations, along with other visits to the Minneapolis Veterans Affairs Medical Center, totaling about 550 unique visits and 7500 encounters.

Based on the population of Hispanic origin (8.5 million) in Texas [33], and an SCI incidence rate of 54 cases per million [32,34], we expect that there are about 432 new cases of SCI per year in Texas. Our UTRGV team has committed to assisting with recruitment and retention strategies in minority groups in the Rio Grande Valley, with a 98% Hispanic population, and where almost 30% live in poverty [35,36].

There are about 200-300 adults with SCI in the Rio Grande Valley: 75-100 treated at UTRGV and >250 treated at the Rehabilitation Institute at the Doctors of the Hospital Renaissance in the Rio Grande Valley. The UTRGV team will involve Hispanic community leaders, adults with SCI, their families, caregivers, and SCI health care workers.

We will also recruit nationwide through regional and national SCI or professional organizations and will post fliers and information on relevant websites and social media. We have successfully recruited participants nationwide in our preliminary remotely delivered Qigong study [14]. Given the prevalence of 308,620 adults with SCI in the United States (with 69% neuropathic pain prevalence) and given our completely remote clinical trial part of the proposal, we have many opportunities to recruit 40 participants with SCI-related neuropathic pain (as well as 40 SCI stakeholders for aim 1), even with an estimated 30% screening failures based on inclusion and exclusion criteria.

We have also taken into account patient and public involvement. Literature shows that adults with SCI, their families, and caregivers who have experience with mind and body approaches are more likely to recommend such approaches compared to health care professionals [37]. Therefore, we have 3 SCI consultants on the team. Our Latina consultant in Minnesota will facilitate communication between the research group and the Hispanic community in Minnesota, whereas the SCI consultant in South Texas will do the same for the Rio Grande Valley region, by educating the team and facilitating the cultural acceptability of Qigong, pain management surveys, and the study protocol (including assessments) for the Hispanic community. Our Veteran SCI consultant has given input on the feasibility of Qigong practice for adults with paraplegia and tetraplegia, and the duration of remote clinical assessments. All SCI consultants will provide input throughout the study and be invited to attend quarterly meetings, where they will be informed about the progress of the study.

### Interventions

Two active interventions are proposed, with different “active ingredients.” More information about the interventions is given below.

The Qigong introduction class will be taught over Zoom by a Spring Forest Qigong-certified instructor. Then, participants will practice Qigong at least 3 times per week for 12 weeks with the “Five Element Healing Movements” online video with streaming access from any computer, iPad, or smartphone device, so participants can practice at home or any location with an internet connection. More details about the content of the Qigong practice can be found in our published protocol paper of the Qigong pilot study in adults with SCI-related neuropathic pain [25]. Participants log in with a unique study identification number and password onto the Spring Forest Qigong website to access the video and practice at least 3 times per week for 45 minutes per session. Their practice (time and duration of access) will be logged automatically and sent to the principal investigator (AVdW), who is level 5 (of 5 levels) in the Spring Forest Qigong curriculum, and a certified practice group leader.

Once a month, the Spring Forest Qigong-certified instructor will do a Qigong session 1:1 with each participant over Zoom to monitor their practice, address questions, demonstrate movements, and give guidance as needed to ensure quality control of the practice. We will use the recording option on the Zoom platform, and the video will be stored on our server, called the University of Minnesota secure box.

The active comparison group will receive a daily text or email message (depending on the choice of the participant) linking them to a 2-minute online Qualtrics survey to answer questions related to their pain intensity level, medications taken that day, activities that helped or hindered the pain, and free text on what they did that day that mattered to them. There are no educational or lifestyle advice components, and they will not receive any guidance on any of the active ingredients given in Qigong (ie, breathing, meditation, and meditative movements). Pain management has been examined through an app in multicenter trials and feasibility studies and is being used in large health care systems in Canada [38].

We will organize weekly (during the intervention) and monthly check-ins (during the 6-month follow-up) to encourage adherence. The Qigong group will also receive a 1:1 session per month with the Qigong instructor to verify the quality of practice and address any questions. The research staff will contact the participant if the pain management surveys are not completed 2 days in a row.

All participants will continue their prescribed or over-the-counter pain medication and maintain their regular standard-of-care health care appointments. However, any current regular (3 times per week or more) mind and body practices, such as Tai Chi, Qigong, or any rehabilitation programs that would influence outcomes, will not be permitted.

The study does not provide compensation for research-related injuries or unexpected urgent care.

### Demographic and Clinical Outcomes

Four types of remotely delivered assessments are used in this study: (1) demographic and SCI-related medical information, (2) feasibility benchmarks, (3) patient-reported outcome measures (questionnaires and EMA surveys), and (4) objective measurements with the commercially available Fitbit Charge 6 smartwatch.

#### Demographic, SCI-Specific Data, and Screening

We will collect National Institute of Neurological Disorders and Stroke – Common Data Elements (NINDS-CDE) SCI-demographic, SCI-relevant general health, and SCI-specific data. We will screen participants with the Mini-Mental State Examination-short version (cutoff score <13/16) [39], and test their ability to perform kinesthetic motor imagery with the Kinesthetic and Visual Imagery Questionnaire (cutoff score <15/25 points) [40]. While imagining body parts affected by SCI may be difficult for participants at baseline, overall impaired kinesthetic imagery is rare: no adult with SCI scored below the cutoff score in our prior studies [14,41].

#### Feasibility Benchmarks

Our feasibility markers are consistent with models and guidelines for intervention development (Table 2) [29,42]. Based on prior publications [43-46], we will assess the following a priori feasibility markers.

**Table 2:** Feasibility benchmarks.

	Feasibility item and details	Benchmark (go/no-go criteria)
Recruitment and enrollment	Assess recruitment rates (screens and enrollment per trimester, sex, and racial outcomes). Evaluate the recruitment rate for complete and incomplete SCI^a^. We aim to recruit 40 participants between mid-year 1 and year 2.	The proportion of potential participants contacted who agreed to participate: 80% is excellent and 70% good. Ratio of complete/incomplete SCI. We estimate recruitment of 30%-40% of adults with complete, 60%-70% with incomplete SCI. Reasons for (not) participating and information collected during focus groups or interviews will help delineate facilitators and barriers to participation, including computer or internet access.
Feasibility and acceptability of the study design	Attrition rates in the Qigong or pain management survey groups.	Proportion of people withdrawing from the interventions. Dropout due to unrelated reasons will count as part of the general attrition. A proportion of 90% is excellent, and 80% good.
Feasibility, adherence, and acceptability of the Qigong practice	Assess intervention adherence, average Qigong practice, at least 3×/week (123 minutes), pain management surveys (daily), and satisfaction with the Qigong practice or pain management surveys. Identify facilitators and barriers to practicing Qigong at home or completing the pain management surveys.	Weekly and monthly check-ins (reporting minutes/week of practice) to identify facilitators and barriers to pain management surveys or practicing Qigong. The Qualtrics website (for pain management surveys) or Spring Forest Qigong website (for Qigong practice) logs time and duration of video access. A Spring Forest Qigong-certified instructor will do a 1:1 Qigong session with each participant via Zoom monthly for quality control and to give guidance. Feasibility: Excellent: >80% participants practice at least 2×/week; good ≥70%. For the pain management survey: completion of surveys at least 5 out of 7 days. Dropout due to lack of interest will be counted as general attrition: 10% dropout is excellent, 20% is good.
Feasibility of adhering to the collection of quantitative measures	% Adherence in completing all assessments during the study and in the monthly follow-up phase until 6 months postintervention. % Adherence to wearing the Fitbit Charge 6 smartwatch and sending the data through their Fitbit app on their smartphone.	Feasibility is ‘excellent’ when none of the Zoom questionnaires, EMA^b^ surveys, or weekly and monthly check-ins are fully missing in >25% of the participants. Excellent adherence: data from the Fitbit app at least 70% of the time and wearing the Fitbit Charge 6 at least 5 out of 7 days/week for at least 7 hours/day (≥80% excellent; ≥70% good).
Program satisfaction	3-item Client Satisfaction Scale [47]. Ask about their intent (and frequency) to continue the Qigong practice or pain management surveys after the 12-week intervention program.	Excellent satisfaction if ≥75% of the participants score above the scale midpoint; and good if ≥70%. Our benchmark is ≥70% of the participants intend to continue the Qigong practice or pain management surveys after 12 weeks.
Program safety	Assess adverse events (IRB^c^+GCP^d^ reporting guidelines) and report adverse events via ACTTION^e^ recommendations [48,49].	Excellent safety: no study-related adverse events; good if there are minimal and mild adverse events (eg, muscle soreness) linked to Qigong practice in max 10% of participants, or any adverse events with the pain management surveys. We will ask about adverse events during the weekly and monthly check-in calls.

^a^SCI: spinal cord injury.

^b^EMA: ecological momentary assessment.

^c^IRB: Institutional Review Board.

^d^GCP: Good Clinical Practice.

^e^ACTTION: Analgesic, Anesthetic, and Addiction Clinical Trial Translations, Innovations, Opportunities, and Networks.

#### Primary Clinical Endpoints

We will use the patient-reported outcome measures of pain intensity PainDETECT [50] and the Numeric Pain Rating Scale [31] for the highest, average (ie, pain experienced most of the time), and lowest pain intensity in the prior week. The Numeric Pain Rating Scale [31] and PainDETECT [50] will be assessed weekly during the Qigong or pain management survey intervention period and then monthly during the 6-month follow-up period.

#### Secondary Clinical Endpoints

To address whole-person health, all secondary outcomes, which are patient-reported outcome measures, will be assessed at 3 time points (baseline, after the intervention period, and at 6 months follow-up). In addition, we will perform EMA assessments 4 times per day for 3 weeks at baseline, during the last 3 weeks of the 12-week intervention, and during the last 3 weeks of the 6-month follow-up. The Fitbit Charge 6 smartwatch is worn all the time during the duration of the study to measure physical and emotional functioning as well as obtain objective measurements on sleep, activity, and other information such as heart rate and stress levels (heart rate variability–based measure).

##### Impact and Type of Pain (NINDS-CDE SCI)

The NINDS-CDE SCI International SCI Pain Basic Data Set Version 3.0 [51] assesses different types of pain, the body location, average pain intensity, and neuropathic pain interference with mood, activity, and sleep. NINDS-CDE SCI–recommended PainDETECT [50] helps identify neuropathic pain phenotypes [52].

##### Physical Functioning

NINDS-CDE SCI Functional Index/Assistive Technology measures function related to basic mobility, self-care, fine motor function, and ambulation [53]. For the Patient Specific Functional Scale, participants rate their ability to achieve 3 self-identified important functional activities or goals [54].

##### Emotional Functioning

Patient Health Questionnaire-2 is used to assess symptoms of depression in adults with SCI [55-58]. We will use the NINDS-CDE recommended SCI-Quality of Life measurement [59] subscales on pain behavior, positive affect, resilience, and ability to participate, to obtain a comprehensive view of the participant’s emotional functioning [59].

##### Global Improvement

The Initiative on Methods, Measurement, and Pain Assessment in Clinical Trials–recommended Patient Global Impression of Change provides a measurement system with scores ranging from 1 to 7 for how a participant evaluates changes in their status, with score 1 reflecting “very much improved,” and gradually decreasing to 4 reflecting “no change” and further down to 7 reflecting “very much worse” [60,61].

##### EMA Surveys

EMA surveys will be sent through text or email: 27 real-time questions total; 10-item Positive and Negative Affect Schedule-Short Form (PANAS-SF) [62], activity, sleep, stress, mind awareness, body awareness, pain interference, and agency (ie, control over life and control over pain) to explore the dynamics of neuropathic pain intensity in daily life [63]. Two cognitive tests will be conducted, with a duration of 30-90 seconds total: (1) attention and set-shifting: number and number/letter sequencing on a 4×4 grid, that is, 1, A, 2, B, 3... (2) spatial working memory test: remembering sequence + location of squares on a 4×4 grid. Providing EMA 4 times per day for 3 weeks is feasible in terms of participant burden, while at the same time providing enough data to perform personal causal modeling.

#### Objective Measurements of Sleep, Activity, and Other Information, Obtained With a Consumer Device, the Fitbit Charge 6 Watch

Our primary data will be physical activity (minutes), heart rate (every second), stress level (heart rate variability–based), and sleep (minutes). Participants will install the Fitbit app with their account and use the OAuth 1.0a protocol on a University of Minnesota website (developed by coauthor KOL) to authorize Fitbit to transfer data directly to our University of Minnesota server. Participants will leave the Fitbit app open in the background so data can be transmitted to our server. In this way, we can monitor that data is being received and that the device is being charged. KOL has used this method successfully in several studies [64,65].

Virtual delivery of questionnaires related to assessments or check-ins will be organized through our secure University of Minnesota Zoom. Participants will be encouraged to complete all scheduled virtual visits and assessments. However, missed sessions will be allowed and recorded and will not be considered protocol deviations. We had 100% adherence to Qigong training, assessments, and weekly check-ins through Zoom in our prior Qigong studies (IRB# STUDY00005656; IRB# STUDY00011997) in those who completed the study. If an EMA survey, pain management assessment, or weekly or monthly check-in is missed, measures will be taken to get the information later. There are no stop criteria for missed sessions or missed testing. The clinical trial will be completely remotely delivered.

### Statistical Analyses

#### Sample Size Calculation

We plan on recruiting 40 SCI stakeholders (aim 1) and 40 adults with SCI-related neuropathic pain (aim 2). Stakeholders not only include adults with SCI but also caregivers and health care professionals and any other stakeholder related to adults with SCI.

Aim 1 requires a qualitative analysis, and thus, power analysis is not calculated.

The sample size for aim 2 of 40 adults with SCI-related neuropathic pain is based on our estimated accrual and enrollment rates from our prior studies on related research. Due to the nature of a feasibility study, power calculations were not conducted. With N=40, we will be able to estimate a rate for the feasibility endpoints, for example, the dropout rate, of 80% with the margin of error of the 95% CI controlled at ±10%. Based on prior literature, the sample size is sufficient to provide reliable estimates of the mean and SD of pain (primary outcome) and other outcomes, to determine the required sample size for future large efficiency studies.

#### Data Analysis

The demographic variables and other baseline characteristics, including sex, race, and ethnicity, will be summarized using descriptive statistics, including mean, median, SD, IQR, minimum, and maximum. Rate parameters for the feasibility markers will be estimated based on the participants who achieved each benchmark. All quantitative variables, including primary and secondary outcomes, will be summarized using descriptive statistics. For longitudinal outcomes, missing data will not be imputed. Mean and SD will be calculated for each outcome at each time point. Means and SDs of across-time changes (eg, pre- vs post-Qigong or pain management survey changes) and correlations will be estimated for all participants, each group, or stratum that will be used for sample size determination in future studies. Mediation analyses will explore potential mediators among variables measured with the Fitbit device for the relation between Qigong practice or pain management surveys and changes in neuropathic pain. Descriptive and feasibility data related to adults with health disparities will be investigated. Missing data will not be imputed. The % missingness will be tabulated for each time point by group, and the mechanism of missingness will be identified.

The qualitative variables (eg, focus groups, interviews, weekly and monthly check-ins) will be recorded, transcribed, and managed using NVivo (Lumivero). Coded units will be sorted into categories and analyzed for recurrent themes or patterns. Emerging categories will be reviewed for alignment with the quantitative findings. These findings will serve to reach conclusions on the feasibility, acceptability, and satisfaction of adults with SCI-related neuropathic pain in study participation and performing Qigong practice or completing pain management surveys. We will also report on conclusions relevant to adults with health disparities.

### Research Team

Our multidisciplinary team has the expertise in all study aspects to successfully carry out these aims. The principal investigator (AVdW) is a NIH-funded new investigator with 27 years of expertise in body awareness research, including Qigong, and 10 years of clinical trial management, including in adults with SCI. She is responsible for the fiscal and research administration. AVdW will oversee the study design, study coordination, data management, regulatory compliance, data collection, and analysis. She will oversee the project and data manager, as well as the research assistants who are blinded to group allocation to perform the assessments. Co-investigator LRM is a SCI clinician and a DoD, NIH, and National Institute on Disability, Independent Living, and Rehabilitation Research–funded researcher with expertise in SCI clinical trial management. Co-investigator LZ is an NIH-funded biostatistician. Co-investigator KOL is an NIH-funded scientist with expertise in using EMA and the Fitbit Charge 6 smartwatch for research. He has built a program, housed on a University of Minnesota server, to download and analyze the data from the Fitbit device. At the University of Minnesota, we will have a program and data manager, experienced in working with remote teams and multiple sites, and a bilingual Latina SCI consultant, as well as a Veteran SCI consultant. Collaborators at the UTRGV are KB, NIH and DoD-funded SCI researcher; BW, SCI clinician neurologist; and GM, a bilingual (Spanish and English) NIH-funded scientist and expert in qualitative research in Hispanic communities. GM has built strong connections with this community and has experience with holding event meetings, focus groups, and interviews to effectively identify facilitators and barriers to health-related study participation and implementation [66,67]. The UTRGV team has committed to helping with the recruitment of adults with SCI in the Rio Grande Valley. They have a bilingual program manager also serving as a Spanish translator if needed, and a bilingual SCI consultant. We have ongoing collaborations with Dr Looft, associate director of Spinal Cord Injuries and Disorders Research at the Minneapolis Veterans Affairs Spinal Cord Injury Center, Minnesota (MN), for recruitment of Veterans, and with Mr Thorson at Essentia Health-Duluth (MN), a rural site for recruitment of adults with SCI.

The principal investigator and program and data manager will meet weekly, and the entire study staff will meet monthly to focus on the progress of the study until project completion. Issues requiring immediate attention across sites will be addressed either by phone, email, Zoom, or in person. The author order of publications will be based on the relative scientific contributions of the principal investigator and key personnel.

### Ethical Considerations

We follow the ethical guidelines of the Declaration of Helsinki. The University of Minnesota’s Institutional Review Board (IRB) approved the study (IRB#STUDY00022415). The study is registered at ClinicalTrial.gov (NCT06140355).

All protocol amendments will be submitted for IRB approval prior to implementation and communication with the participants. If applicable, a new consent form will be signed. New consent forms and protocol amendments will be sent to NCCIH at the time of yearly reporting. Prior to consenting, the consent form will be sent via an email link for review. Eligible participants will have a virtual visit with study staff to review the consent document and ask questions. We will maximize IRB-approved eConsenting and use consent through a legally authorized representative if the participant is physically unable to sign. We will not enroll minors or participants unable to consent due to an altered mental capacity.

We use the University of Minnesota Research Electronic Data Capture (REDCap) database to send out and have participants digitally sign the eConsent forms. REDCap uses a MySQL database via a secure web interface. All IRB rules and HIPAA (Health Insurance Portability and Accountability Act) rules protecting the participants’ rights and confidentiality of records will be followed.

The HIPAA-eConsent forms, electronic medical record information (if applicable), and assessment data will be entered into the password-secured REDCap. EMA surveys and pain management surveys are collected on Qualtrics, and the Qigong practice is logged and sent as a Microsoft Excel file to the principal investigator (AVdW). Virtual delivery of patient-reported outcome measures will be organized through our secure University of Minnesota Zoom. Their responses, including those during the weekly and monthly phone calls, will be entered into the REDCap database. Data on REDCap will be verified by the research staff for completeness and accuracy.

Data integrity and privacy are ensured by attributing a unique study identification number to each participant. Only the principal investigator and approved research staff designees have access to the key, identifying participants with these codes. All data are stored on secure username and password-protected University of Minnesota servers, which are operated by the Academic Health Center’s Information Systems group, and backed up daily, weekly, and monthly. Their servers provide a stable, secure, well-maintained, and high-capacity data storage environment.

Data analysis (including assessments, notes, recordings, and NVivo analysis) is stored on the University of Minnesota secure box, with two-factor authentication. The Fitbit app on their smartphone will transmit data to the Fitbit server, which pushes data to a secure University of Minnesota server (monitored by coauthor KOL). Team members interacting with participants or their data completed the University of Minnesota’s training requirements for the Responsible Conduct of Research, Good Clinical Practice, HIPAA, data security, and study-specific training. They comply with all guidelines and maintain Good Clinical Practice training certificates every 3 years.

After study completion, deidentified data related to the outcomes of the aims of this project will be archived on the Dryad repository. The DOI link will be shared in the peer-reviewed publications together with the publication of the results of this study to ensure sufficient quality to validate and replicate research findings.

Regarding compensation, for aim 1, all participants receive US $50 when they complete either a focus group meeting or an interview. For aim 2, participants will receive a Fitbit that they can keep after the study ends. The participants in the Qigong group will also still have access to the Qigong video after the study has been completed. All participants receive US $200 at the end of the study and an additional US $1 for every EMA survey they have completed at the 3 time points during the study (maximum US $252).

Given that Qigong involves slow, gentle movements in standing, sitting, or lying positions, we do not expect any study-related adverse events from the treatment, similar to our experience in our preliminary studies [14,27]. We also do not expect any study-related adverse events with the pain management surveys. We will monitor adverse events weekly through direct check-in phone calls during the intervention period and monthly during the follow-up period and follow the Good Clinical Practice procedures.

Because there is minimal risk associated with this study, the IRB does not require a Clinical and Translational Science Institute data integrity monitor or data monitoring committee. The principal investigator (AVdW) and LRM (study physician) will determine whether an adverse event is expected or unexpected, if it occurs. An adverse event will be considered unexpected if the nature, severity, or frequency of the event is not consistent with the risk information previously described for the study intervention. Aside from the weekly and monthly check-ins, we will ask participants to reach out to the principal investigator or project and data manager, if (severe) adverse events occur in the period between the calls. We will follow all regulations and protocols as outlined by the University of Minnesota’s IRB should this occur.

Our safety monitoring plan includes an independent medical monitor: Dr Andrew Grande, neurosurgeon at the University of Minnesota, is not associated with this research project and works independently of the principal investigator (AVdW). He is not part of the key personnel involved in this grant, nor has he collaborated or copublished with the principal investigator within the past 3 years. He is qualified to review the patient safety data generated by this study because of his unique expertise as a neurosurgeon.

Dr Andrew Grande will review all reported adverse events semiannually and as needed with the principal investigator (AVdW) to review the number and type of adverse events. The principal investigator will inform Dr Grande promptly of all study-related unanticipated problems involving risk to participants or others, serious adverse events, and all participants’ deaths associated with the protocol, and provide an unbiased written report of the event to the IRB. The medical monitor will comment on the outcomes of the event or problem, and in the case of a serious adverse event or death, comment on the relationship to participation in the study. The medical monitor will also indicate whether he concurs with the details of the report provided by the study investigator. Reports for events determined by either the investigator or medical monitor to be possibly or definitely related to study participation, and reports of events resulting in death, will be promptly forwarded to the University of Minnesota’s IRB and NCCIH.

## Results

We report protocol version 2 and do not anticipate any further changes until trial completion. We registered and posted the trial on ClinicalTrial.gov registration (NCT06140355) on November 20, 2023. The first participant was recruited on August 20, 2024. Study completion is estimated on January 31, 2027. At this time (September 2025), 20 participants have been recruited for aim 1 (no dropouts) and 36 participants were recruited for aim 2, with 8 participants dropping out of the study.

## Discussion

### Anticipated Findings

With this NCCIH-funded feasibility HAPPINESS trial, we will expand on our prior study to consolidate feasibility with a rigorous protocol. We expect that the focus groups and interviews with SCI stakeholders will reveal meaningful and actionable insights regarding facilitators and barriers to Qigong, to all study components, as well as to current health care and neuropathic pain treatments. We expect to gather useful and actionable information on specific problems encountered, and cultural sensitivity to be considered in adults with health disparities (aim 1). We further expect that the study will be feasible, with both interventions being attended and feasibility established for recruitment and enrollment, including the recruitment of 50% of adults with health disparities.

### Limitations

While the completely remote aspect of this study allows for recruitment nationwide and includes rural areas, we are limited to patient-reported outcome measures through Zoom and objective data from the Fitbit. Clinical in-person testing of sensation, movement, and function cannot be performed in this study design, given that it is a fully remote-delivered clinical trial.

### Dissemination Plan

We will present the results of this study at conferences and in peer-reviewed publications. All key personnel, research staff, and stakeholder contributions will be acknowledged. We will use our connections with national SCI advocacy groups and professional associations to disseminate information about the results of the Qigong versus pain management surveys and hold dialogues with stakeholders. The results from this feasibility study will also be used for a federal grant submission for a subsequent effectiveness randomized clinical trial. We will use social media and our Brain Body Mind Lab website platform and other means to educate SCI stakeholders, including health care providers locally and nationally.

### Future Directions

Assessing feasibility is the first step. The results from this study will facilitate a rigorous structure to design large effectiveness studies and facilitate a clear pathway for researchers to investigate Qigong and other mind and body approaches for whole-person health in adults with chronic or neurological disorders, including those with health disparities. Furthermore, once effectiveness is demonstrated, remotely delivered Qigong practice can easily be implemented in clinics, in the community, health care facilities, or at home.

## Appendix

Multimedia Appendix 1 SPIRIT checklist.

## Abbreviations

DoD: US Department of Defense
EMA: ecological momentary assessment
HAPPINESS: Changing the Perceived Pain Intensity in Populations With Spinal Cord Injury and With Health Disparities
HIPAA: Health Insurance Portability and Accountability Act
IRB: Institutional Review Board
M Health: University of Minnesota Health
NCCIH: National Center for Complementary and Integrative Health
NIH: National Institutes of Health
NINDS-CDE: National Institute of Neurological Disorders and Stroke – Common Data Elements
PANAS-SF: 10-Item Positive and Negative Affect Schedule-Short Form
REDCap: Research Electronic Data Capture
SCI: spinal cord injury
SPIRIT: Standard Protocol Items: Recommendations for Interventional Trials
UTRGV: University of Texas Rio Grande Valley

## Data Availability

Data sharing is not applicable to this article as no data sets were generated or analyzed during this study.

## References

[1] Taylor SM, Cheung EO, Sun R, Grote V, Marchlewski A, Addington EL (2019). Applications of complementary therapies during rehabilitation for individuals with traumatic spinal cord injury: findings from the SCIRehab project. J Spinal Cord Med.

[2] (2025). Traumatic spinal cord injury facts and figures at a glance. National Spinal Cord Injury Statistical Center.

[3] (2025). Traumatic spinal cord injury demographics at a glance 2025. National Spinal Cord Injury Statistical Center.

[4] Wiffen PJ, Derry S, Moore RA, Lunn MPT (2014). Levetiracetam for neuropathic pain in adults. Cochrane Database Syst Rev.

[5] Mehta S, McIntyre A, Dijkers M, Loh E, Teasell RW (2014). Gabapentinoids are effective in decreasing neuropathic pain and other secondary outcomes after spinal cord injury: a meta-analysis. Arch Phys Med Rehabil.

[6] Silver JK, Flores LE, Mondriguez González A, Frontera WR (2021). An analysis of the inclusion of women, older individuals, and racial/ethnic minorities in rehabilitation clinical trials. Am J Phys Med Rehabil.

[7] Chalageri E, Vishwakarma G, Ranjan RL, Govindaraj R, Chhabra HS (2021). Effect of Rāja yoga Meditation on Psychological and Functional Outcomes in Spinal Cord Injury Patients. Int J Yoga.

[8] Shem K, Karasik D, Carufel P, Kao M, Zheng P (2016). Seated tai chi to alleviate pain and improve quality of life in individuals with spinal cord disorder. J Spinal Cord Med.

[9] Curtis K, Hitzig SL, Bechsgaard G, Stoliker C, Alton C, Saunders N, Leong N, Katz J (2017). Evaluation of a specialized yoga program for persons with a spinal cord injury: a pilot randomized controlled trial. J Pain Res.

[10] Hearn JH, Cross A (2020). Mindfulness for pain, depression, anxiety, and quality of life in people with spinal cord injury: a systematic review. BMC Neurol.

[11] Tsang WWN, Gao KL, Chan KM, Purves S, Macfarlane DJ, Fong SSM (2015). Sitting tai chi improves the balance control and muscle strength of community-dwelling persons with spinal cord injuries: a pilot study. Evid Based Complement Alternat Med.

[12] Qi Y, Zhang X, Zhao Y, Xie H, Shen X, Niu W, Wang Y (2018). The effect of wheelchair tai chi on balance control and quality of life among survivors of spinal cord injuries: a randomized controlled trial. Complement Ther Clin Pract.

[13] Madhusmita M, Ebnezar J, Srinivasan TM, Mohanty PP, Singh D, Pradhan B (2019). Efficacy of yoga as an add-on to physiotherapy in the management of patients with paraplegia: randomised controlled trial. J Clin Diagn Res.

[14] Van de Winckel A, Carpentier ST, Deng W, Zhang L, Philippus A, Battaglino R, Morse LR (2023). Feasibility of using remotely delivered Spring Forest Qigong to reduce neuropathic pain in adults with spinal cord injury: a pilot study. Front Physiol.

[15] Burnett-Zeigler I, Schuette S, Victorson D, Wisner KL (2016). Mind-body approaches to treating mental health symptoms among disadvantaged populations: a comprehensive review. J Altern Complement Med.

[16] Roth B, Robbins D (2004). Mindfulness-based stress reduction and health-related quality of life: findings from a bilingual inner-city patient population. Psychosom Med.

[17] Beitel M, Genova M, Schuman-Olivier Z, Arnold R, Avants SK, Margolin A (2007). Reflections by inner-city drug users on a Buddhist-based spirituality-focused therapy: a qualitative study. Am J Orthopsychiatry.

[18] Dutton MA, Bermudez D, Matas A, Majid H, Myers NL (2013). Mindfulness-based stress reduction for low-income, predominantly African American women with PTSD and a history of intimate partner violence. Cogn Behav Pract.

[19] Donahue ML, Dunne EM, Gathright EC, DeCosta J, Balletto BL, Jamison RN, Carey MP, Scott-Sheldon LAJ (2021). Complementary and integrative health approaches to manage chronic pain in U.S. military populations: results from a systematic review and meta-analysis, 1985-2019. Psychol Serv.

[20] Madsen C, Vaughan M, Koehlmoos TP (2017). Use of integrative medicine in the United States military health system. Evid Based Complement Alternat Med.

[21] (2019). Review study points to most effective mind-body therapies for PTSD. Office of Research & Development.

[22] Lee C, Crawford C, Hickey A, Active Self-Care Therapies for Pain (PACT) Working Group (2014). Mind-body therapies for the self-management of chronic pain symptoms. Pain Med.

[23] Klein P, Picard G, Baumgarden J, Schneider R (2017). Meditative movement, energetic, and physical analyses of three qigong exercises: unification of eastern and western mechanistic exercise theory. Medicines (Basel).

[24] Kuan S, Chen K, Wang C (2012). Effectiveness of Qigong in promoting the health of wheelchair-bound older adults in long-term care facilities. Biol Res Nurs.

[25] Van de Winckel A, Carpentier S, Deng W, Zhang L, Battaglino R, Morse L (2023). Using remotely delivered Spring Forest Qigong™ to reduce neuropathic pain in adults with spinal cord injury: protocol of a quasi-experimental feasibility clinical trial. Pilot Feasibility Stud.

[26] Huberty J, Eckert R, Gowin K, Mitchell J, Dueck AC, Ginos BF, Larkey L, Mesa R (2017). Feasibility study of online yoga for symptom management in patients with myeloproliferative neoplasms. Haematologica.

[27] Van de Winckel A, Zhang L, Hendrickson T, Lim KO, Mueller BA, Philippus A, Monden KR, Oh J, Huang Q, Sertic JVL, Ruen J, Konczak J, Evans R, Bronfort G (2023). Identifying body awareness-related brain network changes after Spring Forest Qigong™ practice or P.Volve low-intensity exercise in adults with chronic low back pain: a feasibility phase I randomized clinical trial. medRxiv.

[28] (2025). 2025 Federal Poverty Guidelines. ProJusticeMN.

[29] Czajkowski SM, Powell LH, Adler N, Naar-King S, Reynolds KD, Hunter CM, Laraia B, Olster DH, Perna FM, Peterson JC, Epel E, Boyington JE, Charlson ME (2015). From ideas to efficacy: the ORBIT model for developing behavioral treatments for chronic diseases. Health Psychol.

[30] (2017). Research framework. National Center for Complementary and Integrative Health.

[31] Farrar JT, Young JP, LaMoreaux L, Werth JL, Poole MR (2001). Clinical importance of changes in chronic pain intensity measured on an 11-point numerical pain rating scale. Pain.

[32] (2011). Spinal cord injury facts and figures at a glance. J Spinal Cord Med.

[33] Texas Population.

[34] Spinal cord injury statistics. Brain and Spinal Cord Injury Rehabilitation.

[35] Velasco-Mondragon E, Jimenez A, Palladino-Davis AG, Davis D, Escamilla-Cejudo JA (2016). Hispanic health in the USA: a scoping review of the literature. Public Health Rev.

[36] (2022). 2022 QuickFacts: Rio Grande City, Texas. United States Census Bureau.

[37] Rudra RT, Farkas GJ, Haidar S, Slavoski KE, Lokey NE, Hudson TR (2018). Complementary alternative medicine practices and beliefs in spinal cord injury and non-spinal cord injured individuals. J Spinal Cord Med.

[38] Bhatia A, Kara J, Janmohamed T, Prabhu A, Lebovic G, Katz J, Clarke H (2021). User engagement and clinical impact of the manage my pain app in patients with chronic pain: a real-world, multi-site trial. JMIR Mhealth Uhealth.

[39] Cummings JL (1993). Mini-Mental State Examination: norms, normals, and numbers. JAMA.

[40] Malouin F, Richards CL, Jackson PL, Lafleur MF, Durand A, Doyon J (2007). The kinesthetic and visual imagery questionnaire (KVIQ) for assessing motor imagery in persons with physical disabilities: a reliability and construct validity study. J Neurol Phys Ther.

[41] Van de Winckel A, Carpentier ST, Deng W, Bottale S, Zhang L, Hendrickson T, Linnman C, Lim KO, Mueller BA, Philippus A, Monden KR, Wudlick R, Battaglino R, Morse LR (2023). Identifying body awareness-related brain network changes after cognitive multisensory rehabilitation for neuropathic pain relief in adults with spinal cord injury: delayed treatment arm phase I randomized controlled trial. medRxiv. Preprint posted online on February 10, 2023.

[42] Rounsaville BJ, Carroll KM, Onken LS (2001). A stage model of behavioral therapies research: getting started and moving on from stage I. Clin Psychol Sci Pract.

[43] Greenberg J, Popok PJ, Lin A, Kulich RJ, James P, Macklin EA, Millstein RA, Edwards RR, Vranceanu AM (2020). A mind-body physical activity program for chronic pain with or without a digital monitoring device: proof-of-concept feasibility randomized controlled trial. JMIR Form Res.

[44] Greenberg J, Lin A, Zale EL, Kulich RJ, James P, Millstein RA, Shapiro H, Schatman ME, Edwards RR, Vranceanu AM (2019). Development and early feasibility testing of a mind-body physical activity program for patients with heterogeneous chronic pain: the getActive study. J Pain Res.

[45] Winckers ANE, Mackenbach JD, Compernolle S, Nicolaou M, van der Ploeg HP, De Bourdeaudhuij I, Brug J, Lakerveld J (2015). Educational differences in the validity of self-reported physical activity. BMC Public Health.

[46] Carl J, Grüne E, Popp J, Pfeifer K (2020). Physical activity promotion for apprentices in nursing care and automotive mechatronics—competence counts more than volume. Int J Environ Res Public Health.

[47] Attkisson CC, Zwick R (1982). The client satisfaction questionnaire: psychometric properties and correlations with service utilization and psychotherapy outcome. Eval Program Plann.

[48] Smith SM, Wang AT, Katz NP, McDermott MP, Burke LB, Coplan P, Gilron I, Hertz SH, Lin AH, Rappaport BA, Rowbotham MC, Sampaio C, Sweeney M, Turk DC, Dworkin RH (2013). Adverse event assessment, analysis, and reporting in recent published analgesic clinical trials: ACTTION systematic review and recommendations. Pain.

[49] Hunsinger M, Smith SM, Rothstein D, McKeown A, Parkhurst M, Hertz S, Katz NP, Lin AH, McDermott MP, Rappaport BA, Turk DC, Dworkin RH (2014). Adverse event reporting in nonpharmacologic, noninterventional pain clinical trials: ACTTION systematic review. Pain.

[50] Freynhagen R, Baron R, Gockel U, Tölle TR (2006). painDETECT: a new screening questionnaire to identify neuropathic components in patients with back pain. Curr Med Res Opin.

[51] Biering-Sørensen F, Charlifue S, Chen Y, New PW, Noonan V, Post MWM, Rupp R, Vogel L (2023). International spinal cord injury core data set (version 3.0)-including standardization of reporting. Spinal Cord.

[52] Edwards RR, Dworkin RH, Turk DC, Angst MS, Dionne R, Freeman R, Hansson P, Haroutounian S, Arendt-Nielsen L, Attal N, Baron R, Brell J, Bujanover S, Burke LB, Carr D, Chappell AS, Cowan P, Etropolski M, Fillingim RB, Gewandter JS, Katz NP, Kopecky EA, Markman JD, Nomikos G, Porter L, Rappaport BA, Rice ASC, Scavone JM, Scholz J, Simon LS, Smith SM, Tobias J, Tockarshewsky T, Veasley C, Versavel M, Wasan AD, Wen W, Yarnitsky D (2016). Patient phenotyping in clinical trials of chronic pain treatments: IMMPACT recommendations. Pain.

[53] Slavin MD, Ni P, Tulsky DS, Kisala PA, Heinemann AW, Charlifue S, Fyffe DC, Graves DE, Marino RJ, Morse LR, Rosenblum D, Tate D, Worobey LA, Dawson MB, Jette AM (2016). Spinal cord injury-functional index/assistive technology short forms. Arch Phys Med Rehabil.

[54] Westaway MD, Stratford PW, Binkley JM (1998). The patient-specific functional scale: validation of its use in persons with neck dysfunction. J Orthop Sports Phys Ther.

[55] Kroenke K, Spitzer RL, Williams JBW (2003). The Patient Health Questionnaire-2: validity of a two-item depression screener. Med Care.

[56] Krause JS, Saunders LL, Reed KS, Coker J, Zhai Y, Johnson E (2009). Comparison of the patient health questionnaire and the older adult health and mood questionnaire for self-reported depressive symptoms after spinal cord injury. Rehabil Psychol.

[57] Bombardier CH, Richards JS, Krause JS, Tulsky D, Tate DG (2004). Symptoms of major depression in people with spinal cord injury: implications for screening. Arch Phys Med Rehabil.

[58] Spitzer RL, Kroenke K, Williams JB (1999). Validation and utility of a self-report version of PRIME-MD: the PHQ primary care study. Primary Care Evaluation of Mental Disorders. Patient Health Questionnaire. JAMA.

[59] Tulsky DS, Kisala PA, Victorson D, Tate DG, Heinemann AW, Charlifue S, Kirshblum SC, Fyffe D, Gershon R, Spungen AM, Bombardier CH, Dyson-Hudson TA, Amtmann D, Kalpakjian CZ, Choi SW, Jette AM, Forchheimer M, Cella D (2015). Overview of the spinal cord injury-quality of life (SCI-QOL) measurement system. J Spinal Cord Med.

[60] Dworkin RH, Turk DC, Farrar JT, Haythornthwaite JA, Jensen MP, Katz NP, Kerns RD, Stucki G, Allen RR, Bellamy N, Carr DB, Chandler J, Cowan P, Dionne R, Galer BS, Hertz S, Jadad AR, Kramer LD, Manning DC, Martin S, McCormick CG, McDermott MP, McGrath P, Quessy S, Rappaport BA, Robbins W, Robinson JP, Rothman M, Royal MA, Simon L, Stauffer JW, Stein W, Tollett J, Wernicke J, Witter J, IMMPACT (2005). Core outcome measures for chronic pain clinical trials: IMMPACT recommendations. Pain.

[61] Dworkin RH, Turk DC, Wyrwich KW, Beaton D, Cleeland CS, Farrar JT, Haythornthwaite JA, Jensen MP, Kerns RD, Ader DN, Brandenburg N, Burke LB, Cella D, Chandler J, Cowan P, Dimitrova R, Dionne R, Hertz S, Jadad AR, Katz NP, Kehlet H, Kramer LD, Manning DC, McCormick C, McDermott MP, McQuay HJ, Patel S, Porter L, Quessy S, Rappaport BA, Rauschkolb C, Revicki DA, Rothman M, Schmader KE, Stacey BR, Stauffer JW, von Stein T, White RE, Witter J, Zavisic S (2008). Interpreting the clinical importance of treatment outcomes in chronic pain clinical trials: IMMPACT recommendations. J Pain.

[62] Karim J, Weisz R, Rehman SU (2011). International positive and negative affect schedule short-form (I-PANAS-SF): testing for factorial invariance across cultures. Proc Soc Behav Sci.

[63] Pagé MG, Gauvin L, Sylvestre M, Nitulescu R, Dyachenko A, Choinière M (2022). An ecological momentary assessment study of pain intensity variability: ascertaining extent, predictors, and associations with quality of life, interference and health care utilization among individuals living with chronic low back pain. J Pain.

[64] Stevenson BL, Kummerfeld E, Merrill JE (2021). Applying causal discovery to intensive longitudinal data. Proc Mach Learn Res.

[65] Stevenson BL, Kummerfeld E, Merrill JE, Blevins C, Abrantes AM, Kushner MG, Lim KO (2022). Quantifying heterogeneity in mood-alcohol relationships with idiographic causal models. Alcohol Clin Exp Res.

[66] Juengst S, Supnet C, Kew CLN, Silva V, Vega M, Han G, Kelley B, Smith ML, Maestre G (2021). Bilingual problem-solving training for caregivers of adults with dementia: a randomized, factorial-design protocol for the CaDeS trial. Contemp Clin Trials.

[67] Reverol CLP, Gavidia NG, Fernández J, Maestre GE (2016). El uso de las TIC en adultos mayores en Maracaibo [Venezuela]. Rev Cienc Humanas Soc.

